# Preoperative MRI-Based 3D Segmentation and Quantitative Modeling of Glandular and Adipose Tissues in Male Gynecomastia: A Retrospective Study

**DOI:** 10.3390/jcm14217601

**Published:** 2025-10-27

**Authors:** Ziang Shi, Minqiang Xin

**Affiliations:** Department of Aesthetic and Reconstructive Breast Surgery, Plastic Surgery Hospital, Chinese Academy of Medical Sciences, Peking Union Medical College, Beijing 100144, China; ziang_shi@cma.org.cn

**Keywords:** gynecomastia, magnetic resonance imaging, three-dimensional segmentation and reconstruction

## Abstract

**Background:** This study aimed to explore the application value of magnetic resonance imaging (MRI)-based three-dimensional segmentation and reconstruction technology for spatial structural identification and volumetric quantification of glandular and adipose tissues in bilateral gynecomastia (GM) patients undergoing surgical treatment, hoping to provide precise imaging data to support clinical surgical decision-making. **Methods:** A retrospective analysis was performed on preoperative MRI images and general clinical data of 52 patients with bilateral gynecomastia at the patient level (bilateral totals, *N* = 52) who underwent surgical treatment in the Department of Aesthetic and Reconstructive Breast Surgery, Plastic Surgery Hospital of Chinese Academy of Medical Sciences, from March 2023 to September 2024. All images were acquired using a SIEMENS Aera 1.5 T MRI scanner with T1-weighted three-dimensional fat-suppressed sequence (t1_fl3d_tra_spair). Semi-automatic segmentation and active contour modeling (Snake model) using ITK-SNAP 4.0 software were employed to independently identify glandular and adipose tissues, reconstruct accurate three-dimensional anatomical models, and quantitatively analyze tissue volumes. **Results:** The MRI-based three-dimensional segmentation and reconstruction method accurately distinguished glandular and adipose tissues in male breasts, establishing precise three-dimensional anatomical models with excellent reproducibility and operational consistency. Among the 52 patients with bilateral gynecomastia, glandular tissue volume exhibited a markedly non-normal distribution, with a median of 6.11 cm^3^ (IQR, 3.03–12.98 cm^3^). Adipose tissue volume followed a normal distribution with a mean of 1348.84 ± 494.97 cm^3^. The total breast tissue volume also showed a normal distribution, with a mean of 1361.97 ± 496.83 cm^3^. The proportion of glandular tissue in total breast volume was non-normally distributed with a median of 0.50% (IQR, 0.27–1.21%), while the proportion of adipose tissue was also non-normally distributed with a median of 99.50% (IQR, 98.79–99.73%). **Conclusions**: MRI combined with computer-assisted three-dimensional segmentation and reconstruction technology efficiently and accurately achieves spatial identification, three-dimensional modeling, and volumetric quantification of glandular and adipose tissues in patients with bilateral gynecomastia. It objectively reveals the spatial compositional characteristics of male breast tissues. This approach provides precise, quantitative data for clinical decision-making regarding surgical treatment of gynecomastia, featuring robust standardization and strong clinical applicability.

## 1. Background

Gynecomastia (GM) is a common benign breast condition in males characterized primarily by abnormal proliferation of glandular breast tissue, clinically manifesting as unilateral or bilateral breast enlargement often accompanied by varying degrees of adipose tissue accumulation [1]. GM occurs frequently among males across various age groups, with prevalence rates exceeding 50% in adolescents and approximately 40% among elderly men [2]. Although GM generally does not cause significant physiological impairment, the conspicuous breast appearance often results in substantial psychological distress, especially among adolescent and young adult males, who commonly experience anxiety, low self-esteem, and even depression, negatively impacting their quality of life and social adaptability [3]. Therefore, the diagnosis and management of GM have increasingly drawn attention from plastic surgeons, highlighting the critical importance of accurately evaluating the tissue composition and volumetric characteristics of the breast in GM patients during treatment planning.

Currently, clinical diagnosis of GM primarily relies on patient history, physical examination, and imaging studies, with imaging examinations playing a pivotal role. Breast ultrasonography, commonly utilized as the initial screening modality for GM, offers advantages such as being radiation-free, cost-effective, and convenient [4]. However, the accuracy of ultrasound examinations heavily depends on operator expertise, and conventional two-dimensional ultrasound images cannot precisely delineate the spatial anatomy and volumetric characteristics of glandular and adipose tissues. Mammography, frequently employed in screening for breast diseases in females, can identify dense glandular tissues; however, due to the generally thin and small size of male breast tissue and overlapping densities between glandular and adipose components, clear delineation is challenging [5]. Furthermore, the radiation exposure associated with mammography limits its routine application in male breast disorders.

In recent years, Magnetic Resonance Imaging (MRI), with its superior soft-tissue contrast, non-invasiveness, and lack of radiation exposure, has increasingly become an essential imaging modality for diagnosing, differentiating, and preoperative evaluation of GM patients. MRI provides significant advantages, including non-invasiveness, radiation-free imaging, high soft-tissue contrast resolution, and robust three-dimensional visualization, enabling clearer and more accurate spatial anatomical delineation between glandular and adipose tissues [6]. Advanced MRI techniques, such as T1-weighted imaging, fat suppression methods, dynamic contrast-enhanced MRI, and diffusion-weighted imaging, effectively differentiate glandular from adipose tissues and hold substantial value in excluding malignant breast lesions [7,8].

Despite the substantial advantages of MRI in the diagnosis and differential diagnosis of gynecomastia (GM), current MRI studies have predominantly relied on qualitative, two-dimensional imaging assessments and lesion identification, lacking comprehensive three-dimensional (3D) structural and volumetric analysis of glandular and adipose tissues in the male breast [5]. Consequently, clinical treatment decisions often depend heavily on subjective physician judgment without clear, quantitative imaging support. In recent years, MRI-based 3D segmentation and reconstruction technologies have gained widespread application in clinical investigations of neurological diseases and head-and-neck tumors [9,10]. Integrating advanced algorithms such as the active contour (Snake) model, these techniques have demonstrated precise tissue boundary delineation and accurate 3D anatomical reconstruction capabilities [11]. By leveraging MRI-based 3D segmentation and reconstruction methods, it is possible to precisely quantify the spatial distribution and volumes of glandular and adipose tissues in GM patients, providing objective and quantitative metrics to guide diagnosis, inform treatment strategies, and facilitate outcome assessment.

Therefore, the current study employs MRI-based 3D segmentation and reconstruction technology as its core methodological approach to quantitatively analyze the spatial characteristics of glandular and adipose tissues in 52 patients diagnosed with bilateral GM. Specifically, the study first involves the collection of MRI imaging data from clinically diagnosed bilateral GM patients, clearly defining MRI scanning equipment, sequences, and parameters to establish a standardized imaging database. Subsequently, using the ITK-SNAP (version 4.0) software [12], semi-automatic 3D segmentation and reconstruction of breast MRI images are performed to accurately measure glandular and adipose tissue volumes, thereby characterizing the volumetric distribution of these tissues across the patient cohort.

In summary, this study aims to verify the applicability and advantages of MRI-based 3D segmentation and reconstruction technology in volumetric analysis of glandular and adipose tissues among GM patients. The goal is to establish an objective, precise, and quantitative imaging foundation to inform clinical diagnosis and treatment decisions, thereby promoting a transition from subjective clinical judgment toward quantitative and precise evaluation in GM management.

## 2. Materials and Methods

### 2.1. Study Participants

This retrospective study enrolled patients diagnosed with bilateral gynecomastia (GM) who underwent surgical treatment at the Department of Breast Plastic and Reconstructive Surgery, Plastic Surgery Hospital of the Chinese Academy of Medical Sciences, from March 2023 to September 2024. General clinical data and imaging studies were collected retrospectively. Inclusion criteria were as follows: (1) complete and clearly visible preoperative MRI data suitable for subsequent analysis; (2) definitive MRI-based diagnosis of bilateral GM; (3) no prior surgical procedures or trauma involving the breast region; and (4) comprehensive clinical records available. Exclusion criteria included: (1) concurrent malignant breast lesions or other breast tumors; (2) GM secondary to drug use, severe endocrine disorders, or systemic diseases; and (3) incomplete imaging data or inadequate image quality that did not meet analysis requirements. Based on these criteria, 52 male patients aged 16–46 years (mean age, 26.94 ± 7.06 years) were ultimately included in this study. All patients provided informed consent prior to surgery. Patient information and imaging data were anonymized for research purposes.

### 2.2. Clinical Data Collection

Clinical data were retrospectively collected from the 52 bilateral GM patients who explicitly requested surgical treatment at the Department of Breast Plastic and Reconstructive Surgery, Plastic Surgery Hospital of the Chinese Academy of Medical Sciences. All patients provided written informed consent for surgery and routine preoperative MRI as part of standard clinical care. No additional research-specific consent was obtained; instead, retrospective data analysis was performed on fully anonymized datasets in accordance with institutional policies and ethical standards. Clinical data collected included: (1) basic demographic data such as age; (2) details of clinical presentation and medical consultations, including chief complaints (breast enlargement, altered breast appearance, or discomfort) and treatment objectives (cosmetic improvement, symptom alleviation, etc.); (3) diagnostic information, confirming that patients underwent preoperative MRI for definitive diagnosis of bilateral GM, with no history of breast tumors, trauma, prior breast surgery, or relevant medication use; and (4) comprehensive preoperative MRI data that met study quality criteria. Other clinical parameters such as disease duration, anthropometric measures (e.g., BMI, chest circumference), or endocrine profiles were not systematically collected, as the primary aim of this study was methodological—focused on imaging-based volumetric assessment rather than clinical phenotyping.

### 2.3. MRI Imaging Protocol

MRI imaging was uniformly performed in the Department of Radiology at the Plastic Surgery Hospital of the Chinese Academy of Medical Sciences, and all data were anonymized before analysis. Preoperative bilateral breast MRI was acquired using a SIEMENS Aera 1.5 T superconducting scanner (Siemens Healthcare GmbH, Erlangen, Germany) equipped with a multi-channel anterior body array coil combined with the integrated spine matrix. Patients were positioned supine, head-first into the scanner; this position was deliberately adopted because it replicates the intraoperative setting and chest wall geometry required for surgical planning, although it differs from the conventional prone orientation with a dedicated breast coil used in diagnostic breast MRI. Both breasts were symmetrically centered within the coil, and patients were instructed to breathe calmly to minimize motion artifacts. Transverse coverage extended from the anterior sternum to the lateral boundary defined by clinical appearance and examination, and longitudinal coverage included the superior and inferior limits of breast tissue and adjacent superficial structures. Although the field of view necessarily encompassed subcutaneous fat external to the glandular capsule for anatomical orientation, only intramammary fat was segmented and quantified; all extramammary/subcutaneous fat was systematically excluded from volumetric analysis. A T1-weighted three-dimensional fat-suppressed gradient echo sequence (t1_fl3d_tra_spair) with spectral inversion–recovery–based fat suppression was used, providing effective signal suppression of adipose tissue and enhanced gland-to-fat contrast. Sequence parameters were echo time (TE), 2.3 ms; repetition time (TR), 5.0 ms; flip angle, 10° (vendor default); and specific absorption rate (SAR), 0.4008 W/kg; the RF frequency was omitted as it is not required for reproducibility. Resolution settings were field of view, 360 × 360 mm; matrix size, 384 × 384; pixel spacing, 0.9375 × 0.9375 mm; and slice thickness, 5 mm, yielding high in-plane resolution but anisotropic voxels. Thirty contiguous slices were obtained per patient to ensure complete bilateral coverage and enable subsequent three-dimensional segmentation and volumetric analyses. Data were acquired by experienced technologists following a standardized protocol, stored in DICOM format, and managed in a secure anonymized imaging database.

### 2.4. Statistical Analysis

Statistical analyses were performed using SPSS software (version 27.0.1). Normality of quantitative variables was assessed using the Shapiro–Wilk test, with analyses consistently performed on bilateral totals per patient (*N* = 52). Variables demonstrating normal distribution were presented as means ± standard deviations (Mean ± SD), whereas non-normally distributed variables were expressed as medians and interquartile ranges (Median, IQR) [13]. To avoid ambiguity, Shapiro–Wilk results were reported separately for each variable, acknowledging that volumetric measures such as adipose volume approximated normality, while ratio-based variables such as fat proportion exhibited skewed distributions. Statistical significance was defined as a two-sided *p*-value less than 0.05.

## 3. Results

### 3.1. Age Distribution of Participants

**Figure 1:**
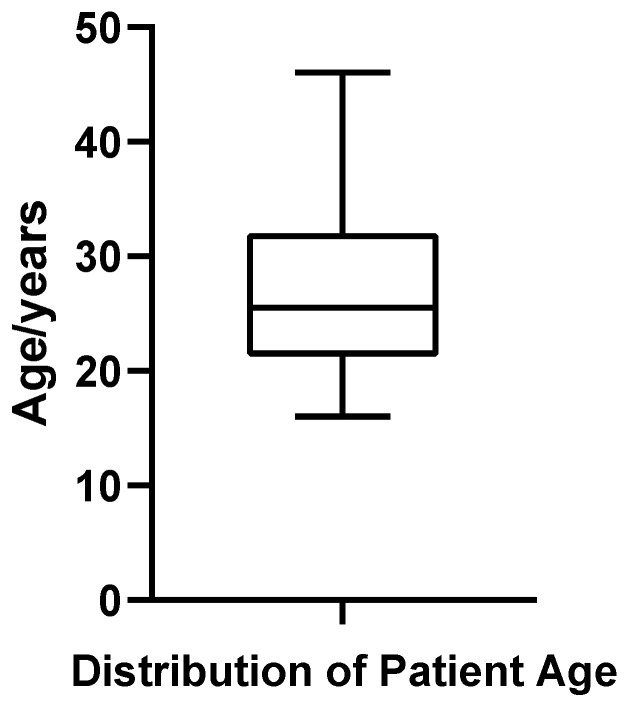
Box plot of patient age in 52 cases of bilateral gynecomastia.

A total of 52 patients diagnosed with bilateral gynecomastia (GM) who underwent surgical treatment at the Plastic Surgery Hospital, Chinese Academy of Medical Sciences, were included in this study. The patient age ranged from 16 to 46 years, with a mean age of 26.94  ±  7.06 years and a median age of 25.5 years. Quartile analysis indicated that the first quartile (Q1) was 21.75 years, the third quartile (Q3) was 32.00 years, and the interquartile range (IQR) was 10.25 years, suggesting that most patients were concentrated between 21.75 and 32.00 years of age (Figure 1). The coefficient of variation (CV%) for age was 26.22%, reflecting relatively high dispersion in patient age distribution. Shapiro–Wilk testing confirmed that age distribution met the criteria for normality (*p* =  0.187).

### 3.2. Application of 3D Segmentation and Reconstruction Technique to MRI Images of Bilateral Gynecomastia Patients

#### 3.2.1. MRI Data Import and Preprocessing

All 52 enrolled patients underwent standardized preoperative MRI examinations, specifically utilizing the T1-weighted three-dimensional fat-suppressed imaging sequence (t1_fl3d_tra_spair). To ensure accuracy in 3D reconstruction and quantitative tissue analysis, this sequence was consistently employed as the foundational imaging dataset for segmentation tasks due to its effective suppression of subcutaneous fat signals and enhanced glandular-adipose tissue contrast, thereby providing a robust basis for subsequent image processing and structural identification.

MRI data were exported in standard DICOM format and imported into the ITK-SNAP (version 4.0) software for 3D segmentation and image analysis. Upon import, all images underwent rigorous checks to confirm structural integrity and spatial continuity, verifying complete coverage of the bilateral breast regions across all 30 axial slices, with clear anatomical boundaries and minimal motion artifacts. Images were preliminarily corrected by adjusting window width and level parameters to optimize tissue contrast, thus ensuring high-quality data for subsequent segmentation tasks.

To maintain consistency and comprehensiveness of the segmentation process, all image segmentation and volumetric calculations were conducted using the entire bilateral breast region of each patient as a single analytic unit, rather than separately analyzing left and right breasts. This approach aligns with the original MRI acquisition method, enhancing structural stability during segmentation and ensuring uniformity throughout the data analysis.

#### 3.2.2. Software Overview and Suitability

ITK-SNAP (version 4.0) was selected to perform three-dimensional segmentation, reconstruction, and volumetric analysis of glandular and adipose breast tissues based on MRI data. ITK-SNAP is a medical imaging software tool built on an open-source image processing platform, widely used for multi-modal imaging data segmentation and three-dimensional modeling. It supports interactive multi-planar visualization of DICOM images, semi-automatic and manual segmentation workflows, precise three-dimensional reconstruction, and voxel-level volume computation. These capabilities make ITK-SNAP particularly suitable for segmentation tasks involving clearly delineated anatomical structures and distinct signal contrast.

The software offers a semi-automatic “seed point plus region-growing” semi-automated segmentation approach combined with the active contour (Snake) model, which ensures stable and precise tissue boundary identification while preserving the original spatial resolution of the images. Its intuitive interface, flexible parameter controls, and real-time visualization capabilities substantially enhance segmentation efficiency and accuracy, especially when analyzing male breast tissues imaged with fat-suppressed MRI sequences.

Given that the T1-weighted three-dimensional fat-suppressed MRI sequence (t1_fl3d_tra_spair) employed in this study exhibits superior glandular-adipose tissue contrast, stable image quality, and clearly defined anatomical layers, ITK-SNAP’s segmentation features and three-dimensional modeling capabilities were highly compatible with the study objectives, fully satisfying the technical requirements for accurate three-dimensional segmentation, reconstruction, and volumetric quantification of glandular and adipose tissues in patients with gynecomastia.

#### 3.2.3. Segmentation Dimensionality and Viewing Strategy

Selecting an appropriate viewing strategy and incorporating cross-plane validation within ITK-SNAP 4.0 software are crucial for achieving accurate structural identification in three-dimensional breast tissue segmentation. The MRI data employed in this study comprised a T1-weighted three-dimensional fat-suppressed sequence (t1_fl3d_tra_spair) with complete spatial information. Images were originally acquired in the axial (transverse) plane, consisting of 30 continuous slices without gaps, ensuring anatomical continuity and spatial coherence.

**Figure 2:**
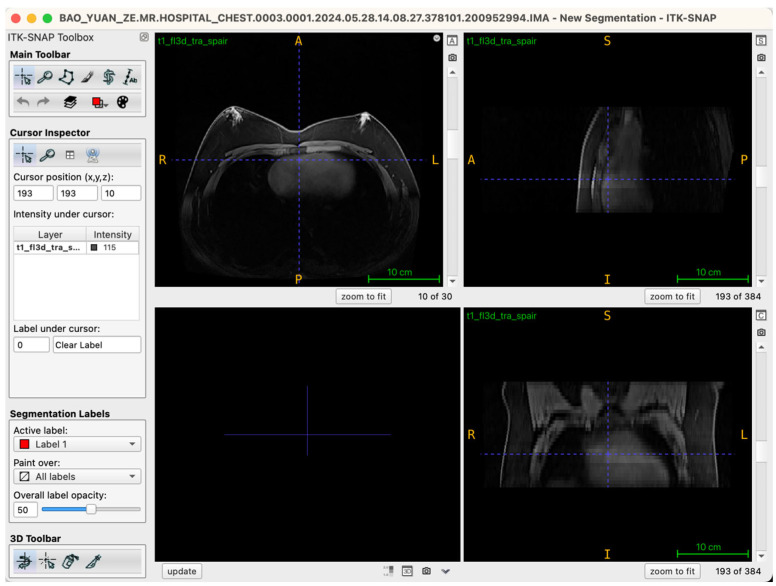
Illustration of the workstation interface utilizing multi-planar reformatting views.

During segmentation, the axial view served as the primary reference plane by default for structural identification and segmentation operations. This choice was based on the clear visualization of breast glandular tissues—which typically present as medium-to-high signal intensity areas—providing clearly discernible boundaries in axial slices. Thus, most seed-point placements, region-growing steps, active contour (Snake) model iterations, and manual corrections were conducted in this plane. To further ensure spatial consistency and anatomical precision of the segmentation boundaries, the Multi-Planar Reformatting (MPR) mode was simultaneously activated throughout the segmentation process, synchronously displaying coronal, sagittal, and axial views (Figure 2). The interactive linkage among these three planes allowed operators to monitor tissue morphology and spatial extension in real-time across different orientations. Particularly in transitional anatomical regions—such as breast margins, pectoral muscle interfaces, and subcutaneous adipose tissue edges—this approach significantly enhanced boundary delineation accuracy. By cross-validating seed-point positions and the progression of segmented boundaries across different views, potential misclassifications, artifacts, or structural discontinuities were promptly identified and rectified during segmentation, thus markedly improving the anatomical fidelity of the final three-dimensional reconstruction. Additionally, this multi-planar visualization approach laid an essential foundation for subsequent reverse rendering-based quality control, allowing operators to verify structural coherence across all imaging planes comprehensively.

#### 3.2.4. Segmentation Targets and Analytical Units

The targets for three-dimensional segmentation from MRI data in this study comprised breast glandular and regional adipose tissues, with the aim of precise structural delineation and volumetric quantification. On the T1-weighted three-dimensional fat-suppressed MRI sequence (t1_fl3d_tra_spair), glandular tissue appeared as regions of medium-to-high signal intensity, primarily concentrated around the nipple level and adjacent slices, with relatively distinct borders and morphologies often characterized as nodular or patchy. In contrast, adipose tissue within the breast area displayed low signal intensity, located around glandular structures extending towards the subcutaneous layer, presenting good continuity but less distinct boundaries. Given the strong tissue contrast and clear visualization provided by this MRI sequence, semi-automatic segmentation methods were particularly well-suited for differentiating and analyzing these tissue types.

In practice, glandular and adipose tissues were independently segmented, each assigned individual segmentation labels. Seed-point placement, region-growing, and active contour evolutions were sequentially performed for each tissue type, followed by volumetric measurements in subsequent analytical steps. This dual-channel segmentation strategy ensured independent and precise structural identification, enabling accurate volumetric calculation and facilitating additional analyses, such as evaluating glandular to total breast volume ratios.

Regarding the analytical unit, the entire bilateral breast region of each patient was treated as the primary unit for segmentation and volumetric analyses. Left and right breast tissues were neither segmented separately nor analyzed independently. Furthermore, individuals with unilateral gynecomastia were excluded from the analysis. Although unilateral male breast enlargement is relatively common clinically, the current study’s focus on standardized three-dimensional structural modeling and volumetric quantification necessitated high standards of image completeness, model closure, and symmetry between sides. Unilateral gynecomastia cases commonly exhibit structural asymmetry, with one side presenting significant glandular proliferation and the contralateral side having negligible glandular tissue, thereby lacking sufficient structural information for robust modeling. From a statistical perspective, despite inherent asymmetry that might exist between bilateral breast structures, the two sides belong to the same individual and thus possess natural biological correlation. Treating bilateral breasts as independent statistical samples would violate independence assumptions, artificially inflating statistical significance and weakening analytic validity due to systematic bias. Clinically, gynecomastia patients typically undergo simultaneous bilateral surgical procedures, and preoperative evaluations inherently rely on comprehensive bilateral breast morphology assessments. Thus, utilizing the combined bilateral breast region as the analytic unit for structural segmentation, three-dimensional modeling, and volumetric analyses aligns seamlessly with MRI acquisition protocols, segmentation methodologies, and clinical practices, thereby ensuring structural completeness, methodological rigor, and robust interpretation of analytical outcomes.

#### 3.2.5. Seed-Point Placement Strategy and Standardization

In the ITK-SNAP 4.0 software platform, seed-point placement represents a critical initial step in the semi-automatic segmentation workflow. By positioning initial seed points within targeted tissue regions and utilizing subsequent region-growing coupled with active contour (Snake) algorithms, precise structural boundary delineation and three-dimensional model construction are achieved. To ensure accuracy, repeatability, and consistency in segmentation, this study established a standardized seed-placement protocol, independently tailored for glandular and adipose breast tissues, incorporating systematic layer-by-layer annotation guided by multi-planar visual validation.

#### 3.2.6. Seed Placement Targets and Principles

Glandular breast tissue and intramammary adipose tissue were segmented independently and reconstructed separately, with designated segmentation labels: “Label 1” for glandular tissue and “Label 2” for adipose tissue. Seed placement followed fundamental principles of clear tissue-specific signal differentiation, distinct anatomical boundaries, and continuous spatial distribution.

Seed points for glandular tissues were preferentially placed at the nipple level and adjacent axial slices where the signal intensity appeared uniform and boundaries clearly defined, thus ensuring central stability of structure identification and coherence of subsequent boundary propagation. For adipose tissues, seed points were strategically placed within low-signal-intensity interstitial regions surrounding and between glandular structures—including areas posterior and adjacent to glandular tissues—explicitly excluding subcutaneous adipose regions to prevent subsequent misclassification of non-target tissues.

#### 3.2.7. Seed-Point Dimensionality and Viewing Strategy

All seed-point placements were performed slice-by-slice on original axial MRI images (transverse views), strictly adhering to a combined two-dimensional slice-based identification and multi-planar reformatting (MPR) approach. Although seed points were placed on two-dimensional slices, simultaneous visualization of coronal and sagittal views allowed operators to verify the three-dimensional continuity and anatomical boundaries of target tissues, effectively preventing inter-slice misplacement or erroneous segmentation in regions of ambiguous tissue differentiation.

#### 3.2.8. Seed-Point Number, Distribution, and Placement Strategy

Each patient’s MRI data consisted of 30 contiguous axial image slices covering the bilateral breast regions comprehensively. During seed-point placement, operators reviewed each slice individually, assessing signal distribution and structural boundaries to adaptively determine optimal seed-point numbers and distribution patterns according to actual glandular and adipose tissue volumes and morphologies.

**Figure 3:**
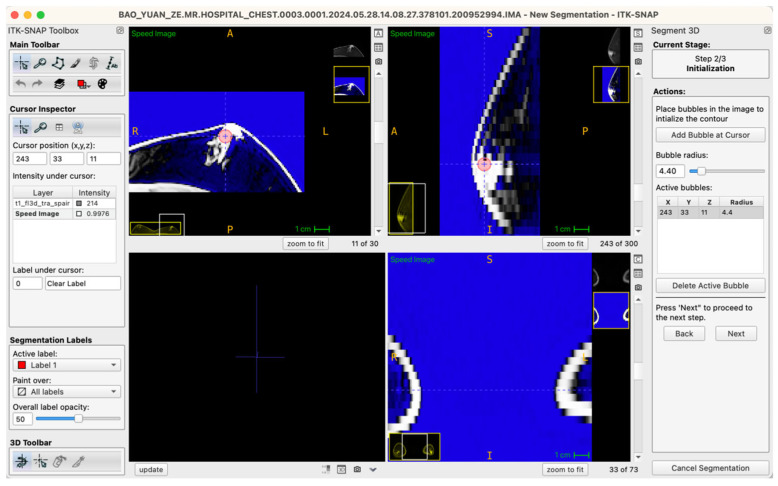
Example workstation interface illustrating glandular tissue seed-point placement on an axial MRI image (seed-point radius set at 4.4 pixels in this instance).

Generally, glandular tissue seed points were positioned within approximately five to six slices centered at the nipple level, typically involving five to ten seed points per case. For larger or irregularly shaped glandular regions, a “central-peripheral collaborative strategy” was employed—establishing core seed points within dense central areas while placing supplementary seed points at peripheral tissue boundaries to enhance segmentation stability and edge definition. To ensure balance between local anatomical precision and overall segmentation accuracy, the radius of seed points was uniformly set within a range of 3.0–6.0 pixels, a parameter range validated through preliminary experiments to effectively cover target structures without excessive expansion, thus ensuring accurate and stable boundary propagation (Figure 3).

Adipose tissue seed points were primarily positioned in the low-signal-intensity regions within or adjacent to glandular tissues, explicitly excluding subcutaneous fat and the anterior border of the pectoral muscle. The seed placement extended across all structural levels within the breast region exhibiting typical adipose tissue signals to ensure comprehensive structural identification.

#### 3.2.9. Integrated Bilateral Seed Placement Strategy

Since the analytic unit in this study was defined as the entire bilateral breast region of each patient, seed-point placement similarly followed an integrated bilateral strategy without separate left-right segmentation or independent operations. Seed points covered all relevant bilateral breast tissues comprehensively, ensuring spatial completeness and consistency in structural identification during model generation, thereby avoiding potential statistical bias arising from artificial separation of bilateral breast analyses.

#### 3.2.10. Seed-Point Verification and Quality Control Measures

All seed-point placements adhered strictly to standardized operational procedures. After initial seed placement, positions and tissue assignments were meticulously reviewed using ITK-SNAP’s slice-navigation capabilities. Real-time monitoring via synchronized multi-planar visualization allowed operators to immediately confirm accurate seed-point localization within target tissues and to detect any inadvertent boundary crossing or inclusion of non-target structures.

If ambiguous boundaries or signal transition regions resulted in potential misclassification, omissions, or unstable segmentation contours, seed points were repositioned and revisions documented explicitly, ensuring transparent segmentation pathways and model structural completeness. Only after confirming complete and appropriately distributed seed points for both glandular and adipose tissues did the workflow proceed to subsequent region-growing and active contour segmentation phases.

#### 3.2.11. Snake Parameter Settings and Segmentation Workflow

Building upon the initial structural identification, this study employed the Active Contour Model (ACM)—commonly referred to as the “Snake” algorithm—embedded within ITK-SNAP 4.0 to accomplish fully three-dimensional, automated segmentation and modeling of glandular and intramammary adipose tissue. This algorithm leverages a biomimetic dynamical approach, constructing a deformable contour that evolves within the image gradient field. Throughout the process, the contour dynamically integrates edge attraction, contour smoothness, and local intensity variation, enabling it to converge upon and closely adhere to true tissue boundaries—even in settings with limited signal contrast or gradual tissue transitions. In the context of male breast MRI, this approach facilitates reliable discrimination between glandular and adipose compartments, increases segmentation efficiency, and minimizes human operator bias.

ITK-SNAP 4.0 implements the Snake algorithm with an automated parameter adjustment mechanism. Based on the user-defined seed regions, native image intensity distributions, and local gradient variations, the software dynamically calibrates the segmentation parameters, permitting standardized, reproducible boundary evolution with no need for manual parameter tuning. The three principal Snake control parameters are curvature (regulates contour smoothness and prevents edge fragmentation), propagation (controls the expansion rate and envelope of the evolving contour), and advection (enhances adherence to image gradient boundaries). This automation ensures consistent, repeatable segmentation performance across all cases under a unified software environment, well-suited for standardized, high-throughput MRI segmentation studies.

**Figure 4:**
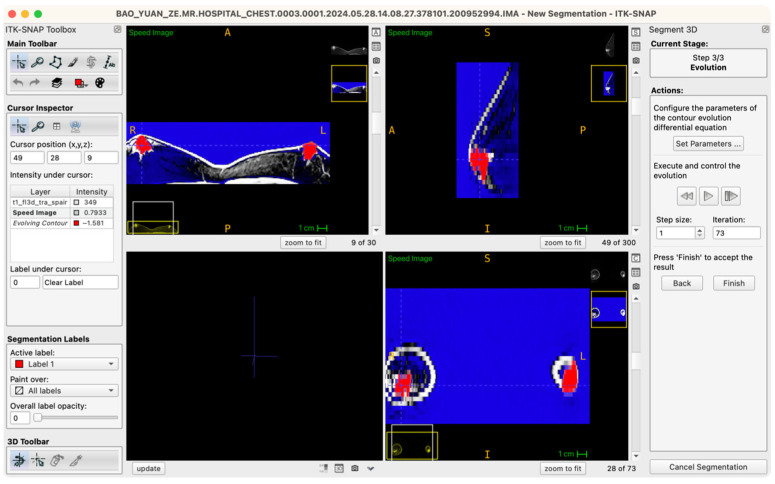
Workstation schematic of glandular tissue segmentation evolution.

The procedural workflow is as follows: after the seed points are placed and labels assigned, the operator enters the “Segment 3D” module and activates the “Run Snake” function. The software then assesses local image intensity in proximity to seed points, executing localized contour evolution. The segmentation process is visualized in real time within a 3D preview window, where the operator can observe contour convergence, boundary accuracy, and structural fidelity relative to the intended tissue morphology. Importantly, the Snake algorithm is executed independently for glandular (Label 1) and adipose (Label 2) tissues, ensuring mutual exclusivity and clarity of boundaries (Figure 4).

Following model generation, ITK-SNAP’s synchronized multi-planar viewing functionality is used for comprehensive quality review. Segmentation boundaries are inspected, slice-by-slice, in axial, sagittal, and coronal planes to confirm precise anatomical adherence and to identify any instances of over-segmentation (“false positives”) or under-segmentation (“missed tissue”). If incomplete contours, boundary drift, or spurious segmentations are detected, the operator can iteratively adjust seed-point distribution and rerun the Snake segmentation until continuous, well-conformed, and topologically accurate models are achieved.

To ensure spatial consistency and segmentation reliability across cases, the entire pipeline was implemented on identical workstation hardware, the same ITK-SNAP software version, and a unified image-processing protocol. All cases were segmented by a single operator with standardized training, with each critical workflow juncture subject to structured review. This combination of automated parameter tuning and stringent quality control enabled standardized recognition of glandular and adipose tissues in all 52 cases, providing a robust anatomical foundation for subsequent volumetric extraction, ratio computation, and 3D visualization analyses.

To further validate the anatomical independence of intramammary and subcutaneous adipose tissue and to ensure exclusion from the model, we placed independent seed points within the subcutaneous fat layer beyond the glandular capsule for every case, generating a separate segmentation rendered concurrently with the glandular/adipose model. Results consistently demonstrated that subcutaneous adipose tissue formed a contiguous, low-signal, band-like distribution external to the glandular envelope, while intramammary fat appeared as discrete, reticulated deposits interspersed within glandular tissue—distinct in both spatial continuity and morphology. This strategy not only enabled effective identification and exclusion of subcutaneous adipose tissue but also ensured that clinicians without a background in breast surgery could readily appreciate structural boundaries and tissue demarcations when interacting with the 3D models.

Collectively, this three-dimensional visualization approach significantly enhances model interpretability and serves as a direct visual validation mechanism, supporting the anatomical and clinical reliability of our tissue classifications. It also establishes a visually rigorous foundation for subsequent result presentation, academic communication, and educational deployment, greatly advancing the explanatory power, reproducibility, and translational value of advanced 3D segmentation methodologies.

#### 3.2.12. Three-Dimensional Reconstruction and Volume Quantification

##### Model Reconstruction Workflow

**Figure 5:**
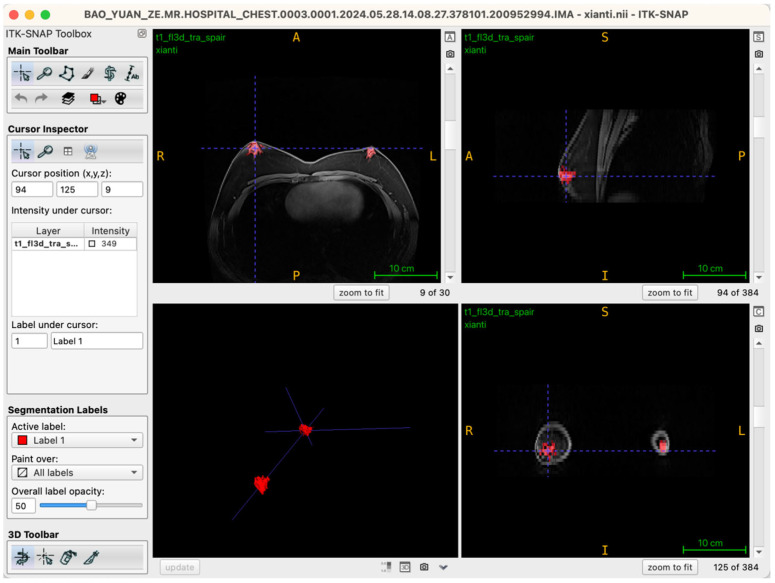
Workstation interface showing the completed 3D reconstruction of bilateral glandular tissues.

Following the completion of three-dimensional segmentation of glandular and intramammary adipose tissues, three-dimensional surface reconstruction was performed using the built-in visualization module of ITK-SNAP 4.0. Based on the label maps generated during segmentation, the software automatically created surface meshes for each labeled structure, rendering the spatial morphology of glandular and adipose tissues in three-dimensional coordinates. This reconstruction process was conducted directly on high-resolution DICOM-format MRI datasets, with the original matrix size preserved at 384 × 384 pixels, in-plane resolution of 0.9375 mm × 0.9375 mm, and a slice thickness of 5 mm across 30 contiguous slices. By maintaining these native imaging parameters throughout the modeling process, anatomical continuity and structural fidelity were ensured (Figure 5).

**Figure 6:**
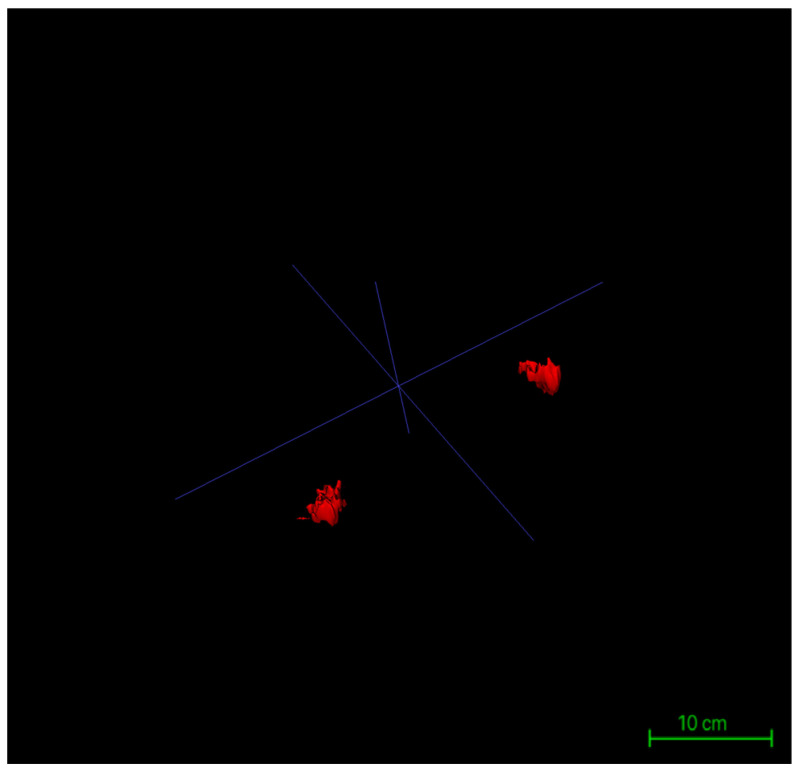
Three-dimensional reconstruction models of bilateral glandular tissues in a representative patient.

Surface rendering was implemented using the Marching Cubes algorithm, the default method in ITK-SNAP, which converts each labeled region into a polygonal mesh. Glandular and adipose tissues were visualized with distinct color codes corresponding to their label IDs, and the resulting models could be rotated, zoomed, and sectioned interactively within the 3D viewer. Following model generation, the reconstructed structures were rigorously verified using synchronized axial, sagittal, and coronal views. Operators examined the alignment of each three-dimensional surface boundary against its corresponding two-dimensional slices to ensure spatial consistency, structural closure, and boundary integrity. This step provided the foundational quality assurance necessary for subsequent volumetric analysis and image-based quantitative assessments (Figure 6).

##### Volume Quantification and Data Standardization

**Figure 7:**
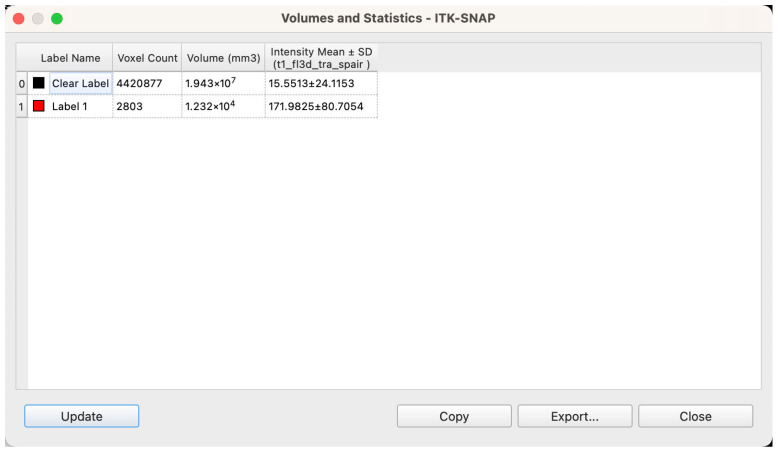
Workstation interface showing voxel-based calculation of spatial volume following segmentation.

Upon completion of segmentation and model reconstruction, volumetric analysis was conducted using ITK-SNAP’s integrated “Label Statistics” module. For each labeled structure, the software automatically calculated total volume based on the number of enclosed voxels and the known voxel size (Figure 7). Prior to export, all label boundaries were manually inspected within the 3D viewer to confirm structural completeness and appropriate segmentation coverage. Volume calculations were based on the native voxel dimensions of the MRI scans—0.9375 mm × 0.9375 mm in-plane resolution with a slice thickness of 5 mm—resulting in a voxel volume of 0.0043945 cm^3^. The following parameters were extracted for each patient: volume of bilateral glandular tissues (Label 1), volume of intramammary adipose tissue (Label 2), total breast region volume (sum of the two), and the relative proportions of glandular and adipose tissue within the total volume, expressed as percentages.

To ensure consistency and facilitate downstream statistical analysis, all volumetric data were exported in CSV format with standardized variable names: Gland_Volume, Fat_Volume, Total_Volume, Gland_Ratio, and Fat_Ratio. All values were converted to cubic centimeters (cm^3^) and rounded to two decimal places to enhance comparability across cases. After export, all datasets were manually validated, including cross-verification with three-dimensional reconstructions, label assignment consistency, and outlier detection. The final dataset was curated in Excel format to support subsequent statistical testing and graphical visualization.

### 3.3. Validation of Segmentation Consistency and Pathways for Reproducibility Control

To ensure the robustness and reliability of three-dimensional segmentation and reconstruction across varying operational contexts, this study implemented a comprehensive verification and reproducibility control framework grounded in a standardized workflow. This framework encompassed four core dimensions: protocol standardization, repeatability testing, inter-operator cross-validation, and full procedural archiving. Together, these components were designed to enhance operational stability, research reproducibility, and future scalability across institutions.

At the foundation of this framework lies a meticulously developed Standard Operating Procedure (SOP), aimed at minimizing operator subjectivity throughout the segmentation process. The SOP covers all critical steps, including DICOM image importation protocols, window width and level settings, multi-planar visualization synchronization, seed-point placement strategies, and active contour (Snake) parameter specifications. All segmentation procedures were performed independently by a single operator who had received formal training in ITK-SNAP to eliminate inter-operator variability. Each case was accompanied by a complete procedural record, including operational logs, stepwise annotations, and tri-planar screenshots of key anatomical slices, ensuring that every model was fully traceable and audit-ready.

To evaluate intra-operator repeatability, a subset of 10 cases was randomly selected from the 52-patient cohort for temporal replication. The same operator, blinded to previous outcomes, re-segmented each case one week after the initial procedure. The Dice Similarity Coefficient (DSC) was used to quantify the spatial overlap between the two segmentation outputs. The average DSC values were 0.931 for glandular tissue and 0.954 for adipose tissue, indicating excellent spatial concordance (DSC > 0.90). These results demonstrated that under standardized conditions, consistent and reliable structural models could be generated by the same operator across different time points.

To further test inter-operator consistency, five additional cases were independently segmented by a second operator with medical background who had undergone identical ITK-SNAP training. Both operators followed the same SOP protocol. Comparative analysis of the volumetric segmentation outputs revealed no statistically significant differences between the two sets of results (*p* > 0.05), with all DSC values exceeding 0.92. This confirmed that, given clear anatomical signal characteristics and a rigorously defined operational protocol, the segmentation workflow exhibited high reproducibility and minimal operator-dependent variability.

In the interest of transparency and quality assurance, all final models were subjected to synchronized multi-planar verification. Each three-dimensional reconstruction was cross-checked against the corresponding axial, sagittal, and coronal slices to ensure boundary closure, structural completeness, and label integrity. Representative 3D renderings and annotated key slice screenshots were exported for every case and archived as quality control documentation.

In summary, this study established a rigorous consistency validation system for MRI-based three-dimensional segmentation of breast tissues by integrating standardized procedural control, intra- and inter-operator reproducibility testing, and systematic result archiving. This framework not only reinforces the credibility and stability of our findings but also provides a scalable quality control model for future segmentation studies across external clinical and research settings.

### 3.4. Volumetric Characteristics of Glandular and Adipose Tissues in Bilateral Gynecomastia Patients

#### 3.4.1. Quantitative Analysis of Glandular, Adipose, and Total Breast Volumes

To comprehensively assess the volumetric characteristics of breast tissue in patients with bilateral gynecomastia, we conducted a detailed quantitative analysis of glandular volume, adipose volume, and total regional breast volume across all cases. All volume parameters were derived from three-dimensional reconstructions generated using ITK-SNAP 4.0 and processed statistically using SPSS version 27.0.1. Normality tests and descriptive statistics were employed to characterize distributional patterns, variability, and inter-individual differences. For visualization, boxplots were constructed for each parameter. The central line within each box represents the median, the box edges correspond to the interquartile range (IQR), and the whiskers extend to values within 1.5 × IQR. Individual dots denote observations outside this range, which are considered outliers. All plots are based on the full analytic cohort (*N* = 52, bilateral totals).

##### Glandular Volume

**Figure 8:**
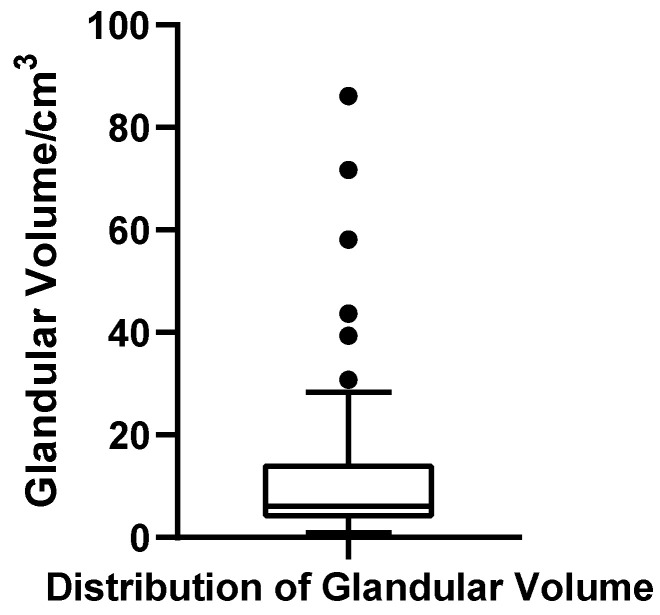
Box plot of glandular volume in 52 patients with bilateral gynecomastia (bilateral totals, *N* = 52).

The glandular volume ranged from 0.92 to 86.13 cm^3^ across the cohort of 52 patients, with a mean of 13.12 cm^3^ (SD = 17.54 cm^3^) and a median of 6.11 cm^3^. The first quartile (Q1) and third quartile (Q3) were 3.03 cm^3^ and 12.98 cm^3^, respectively, yielding an interquartile range (IQR) of 9.95 cm^3^. The coefficient of variation (CV) was 133.7%, indicating substantial dispersion in glandular development. The Shapiro–Wilk test revealed a significantly non-normal distribution (W = 0.637, *p* < 0.001, *N* = 52), with a pronounced right-skew. Based on the conventional criterion for identifying extreme high values (greater than Q3 + 1.5 × IQR, i.e., >27.91 cm^3^), six patients were classified as outliers, suggesting a subgroup with marked glandular hypertrophy. A boxplot was constructed to visually illustrate the wide spread and presence of extremes in this parameter (Figure 8).

##### Adipose Volume

**Figure 9:**
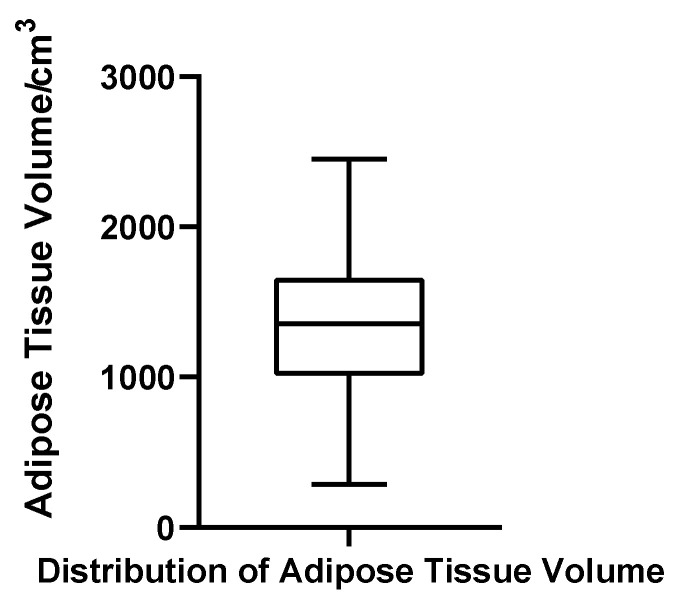
Box plot of adipose tissue volume in 52 patients with bilateral gynecomastia (bilateral totals, *N* = 52).

Adipose tissue volume ranged from 287.28 to 2450.69 cm^3^, with a mean of 1348.84 cm^3^ (SD = 494.97 cm^3^) and a median of 1356.38 cm^3^. The quartile boundaries were Q1 = 1043.75 cm^3^ and Q3 = 1667.08 cm^3^, with an IQR of 623.33 cm^3^ and a CV of 36.7%. The Shapiro–Wilk test indicated that adipose volumes were normally distributed (W = 0.982, *p* = 0.611, *N* = 52), and no statistical outliers were detected. These results suggest that, unlike glandular tissue, adipose volume in gynecomastia patients exhibits a relatively stable and symmetric distribution (Figure 9).

##### Total Breast Volume

**Figure 10:**
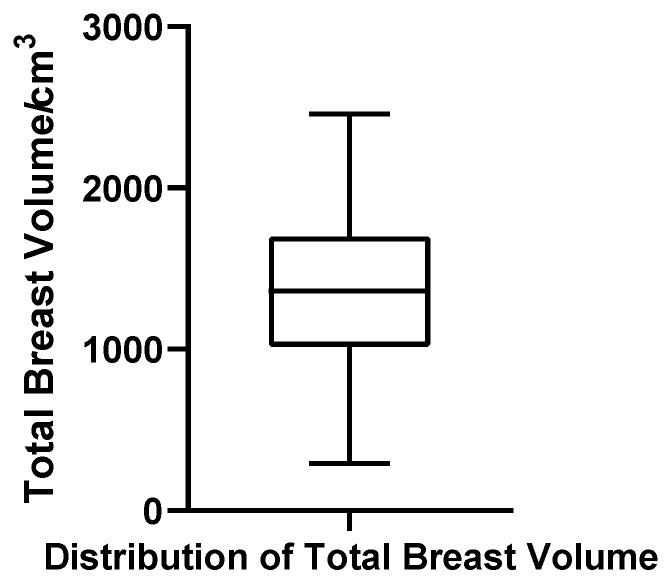
Box plot of total breast volume in 52 patients with bilateral gynecomastia (bilateral totals, *N* = 52).

Total breast volume, defined in this study as the sum of glandular tissue and intramammary fat (excluding subcutaneous/extramammary fat), ranged from 291.15 to 2459.00 cm^3^. The mean and median were 1361.97 cm^3^ and 1360.50 cm^3^, respectively, with Q1 = 1055.68 cm^3^, Q3 = 1712.43 cm^3^, IQR = 656.75 cm^3^, and CV = 36.5%. The Shapiro–Wilk test supported a normal distribution (W = 0.984, *p* = 0.683, *N* = 52), and no outliers were observed. These findings reinforce the notion that total breast volume, dominated by adipose tissue, follows a predictable and homogeneous pattern across patients (Figure 10).

In summary, the volumetric data demonstrate marked inter-individual heterogeneity in glandular development, with glandular volume showing significant non-normality and the presence of extreme values. In contrast, adipose volume and total breast volume followed near-normal distributions with moderate dispersion, reflecting more uniform tissue accumulation patterns. These contrasting patterns underscore the clinical heterogeneity of glandular proliferation in gynecomastia and highlight the need for individualized structural assessment in surgical planning.

#### 3.4.2. Proportional Composition of Glandular and Adipose Tissue Within the Male Breast

To elucidate the structural composition of male breast tissue in gynecomastia, we further analyzed the relative contributions of glandular and adipose components. Based on volumetric data obtained from three-dimensional segmentation and modeling in 52 bilateral gynecomastia patients, we calculated the proportion of glandular volume to total breast volume (Gland Ratio) and the proportion of adipose volume to total breast volume (Fat Ratio). These ratios were used to assess the compositional variability across the study population.

##### Gland Ratio

**Figure 11:**
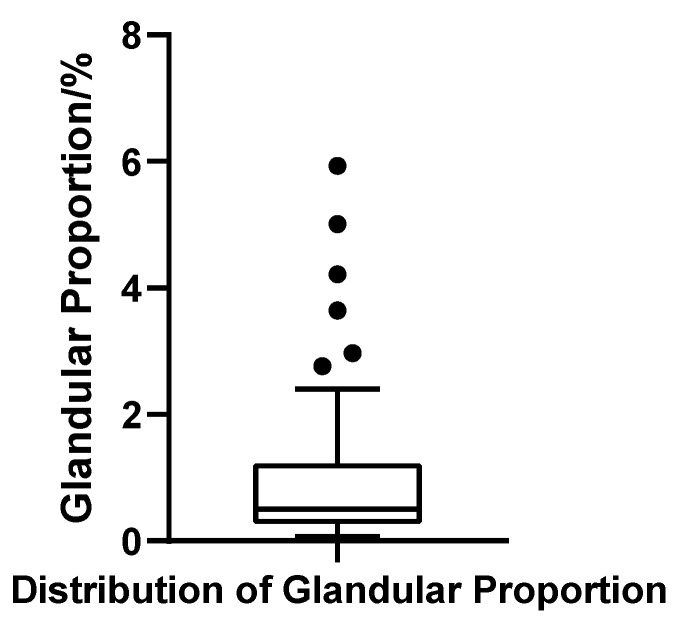
Box plot of glandular tissue proportion in 52 patients with bilateral gynecomastia (bilateral totals, *N* = 52).

The glandular volume ratio varied widely across patients, ranging from 0.07% to 5.93%, with a mean of 1.04% (SD = 1.28%) and a median of 0.50%. The interquartile range (IQR) was 0.94%, with Q1 = 0.27% and Q3 = 1.21%, indicating substantial heterogeneity in glandular contribution. The coefficient of variation (CV) reached 123.0%, further underscoring the marked inter-individual variability. The Shapiro–Wilk test confirmed a strong deviation from normality (W = 0.703, *p* < 0.001, *N* = 52), reflecting a pronounced right-skewed distribution. Applying the outlier criterion (Q3 + 1.5 × IQR), six patients were identified with gland ratios exceeding 2.63%, suggesting significant glandular hypertrophy in a distinct subgroup (Figure 11).

##### Fat Ratio

**Figure 12:**
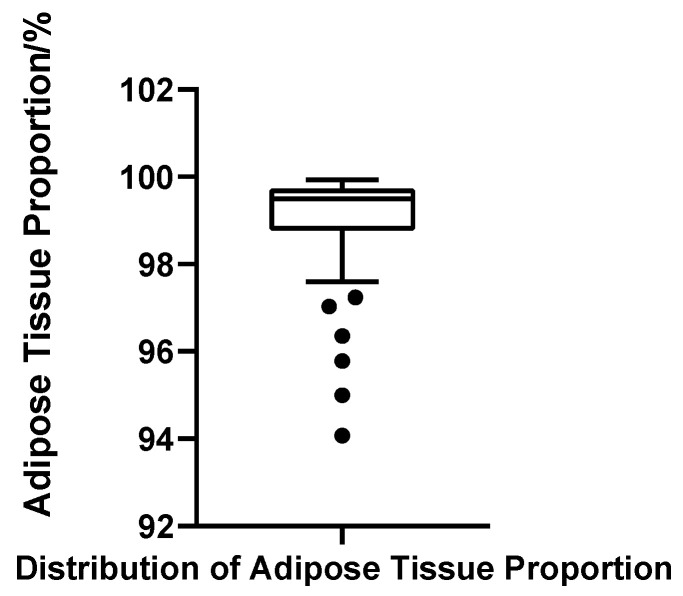
Box plot of adipose tissue proportion in 52 patients with bilateral gynecomastia (bilateral totals, *N* = 52).

Adipose tissue represented the predominant component of the male breast region in all cases, with fat volume ratios ranging from 94.07% to 99.93%. The mean fat ratio was 98.96% (SD = 1.28%), and the median was 99.50%, with Q1 = 98.79% and Q3 = 99.73% (IQR = 0.94%). The CV was 1.30%, indicating minimal variability and a tightly clustered distribution. Although the Shapiro–Wilk test again suggested non-normality (W = 0.703, *p* < 0.001, *N* = 52), this deviation was primarily due to a minority of cases with markedly low fat ratios, which skewed the overall symmetry. Using the threshold of Q1 − 1.5 × IQR (97.37%), six patients were identified with disproportionately low fat content; notably, these cases largely overlapped with those showing elevated gland ratios (Figure 12).

In summary, the male breast in gynecomastia is predominantly composed of adipose tissue, while glandular tissue constitutes only a small fraction. The relative contribution of glandular tissue nevertheless varied considerably across individuals, underscoring the structural heterogeneity of gynecomastia. This variability was also reflected in distributional patterns: absolute volumetric measures such as adipose and total breast volume tended to approximate normality, whereas ratio-based indices showed marked departures from it. The non-normality of glandular and fat ratios stems from their bounded range (0–100%) and the fact that glandular tissue was generally small compared with adipose, which led to clustering of values near the extremes (gland ratio close to 0%, fat ratio close to 100%) and pronounced skew. As gland ratio and fat ratio are exact mathematical complements, they necessarily produced identical Shapiro–Wilk results, a reflection of linear dependency rather than a reporting inconsistency. Taken together, these findings emphasize both the biological variability of gynecomastia and the importance of individualized, imaging-based assessment to inform surgical planning and clinical decision-making.

## 4. Discussion

In this study, we conducted a comprehensive volumetric assessment of the male breast in 52 patients with bilateral gynecomastia who were scheduled to undergo surgical correction. Using MRI-based three-dimensional segmentation and reconstruction, we generated anatomically continuous models of both glandular and adipose tissues, allowing for precise quantification of their volumetric features.

One of the most striking findings was the overwhelmingly low proportion of glandular tissue within the total breast volume in this surgical cohort. The average glandular ratio was only 1.04%, with a median of 0.50%, underscoring a predominant pattern of fatty proliferation. Notably, over 90% of the patients demonstrated glandular contributions of less than 3%, indicating that the majority of cases requiring surgery were characterized by fat-dominant hypertrophy rather than extensive glandular overgrowth. This finding challenges the conventional reliance on subjective intraoperative impressions or two-dimensional imaging assessments and underscores the value of objective structural quantification in preoperative planning [14].

Beyond the anatomical insights, this study introduces a methodological advancement in the imaging evaluation of gynecomastia. The application of a high-resolution T1_FL3D_TRA_SPAIR sequence, combined with semi-automated, multi-label segmentation using ITK-SNAP software, enabled us to independently delineate glandular and adipose tissues and extract their respective volumes with high fidelity. The resulting workflow is not only precise and efficient but also traceable and reproducible—features critical for both clinical application and future research. This framework provides a scalable template for quantitative structural assessment in other morphologically complex soft-tissue regions.

Importantly, our findings are grounded in a highly selected clinical population—namely, patients seen at the Department of Aesthetic and Reconstructive Breast Surgery of the Plastic Surgery Hospital, Chinese Academy of Medical Sciences, who had opted for surgical intervention. This group does not represent the full spectrum of gynecomastia but rather a subset with substantial structural abnormalities, significant subjective distress, and clear surgical indications. In the context of clinical decision-making, such patients are highly representative of those who truly benefit from preoperative anatomical modeling. Although no formal sample size calculation was performed, the inclusion of 52 bilateral surgical cases provided more than adequate power for the descriptive and reproducibility-focused objectives of this study. Because the aim was not hypothesis testing but rather detailed structural quantification and methodological validation, this cohort size was sufficient to reveal distributional patterns, capture inter-individual heterogeneity, and support robust segmentation repeatability.

Compared with those with mild or incidental gynecomastia, the surgical cohort typically presents with pronounced morphological alterations, including visibly enlarged breast volume, aberrant tissue distribution, and aesthetically concerning deformities. For these patients, preoperative structural quantification offers critical value in tailoring surgical techniques and managing expectations. Furthermore, most had undergone endocrine evaluation prior to surgery, making their imaging features more reflective of the disease’s morphological endpoint. As such, the observed glandular and adipose composition likely mirrors intraoperative realities and the primary determinants of postoperative contour, reinforcing the clinical utility of this imaging-based approach.

In clinical practice, the histopathological composition of gynecomastia (GM) is notably heterogeneous. Although an increase in breast volume is a shared manifestation across cases, the internal tissue architecture—specifically the balance between glandular and adipose components—varies substantially among individuals [15]. By leveraging three-dimensional segmentation and modeling, the present study offers a precise quantification of this gland-fat ratio in a surgical GM cohort, thereby uncovering a predominant pattern of “fat-dominant structure” in bilateral cases. This finding not only provides objective confirmation of prior observations largely based on subjective or two-dimensional imaging interpretations but also establishes a foundational framework for structural subtyping in GM.

From a surgical standpoint, the relative proportion of glandular versus adipose tissue plays a pivotal role in determining the optimal approach [16]. In patients with minimal glandular hypertrophy, liposuction alone may suffice to restore normal contour. Conversely, in cases characterized by marked glandular proliferation, more targeted excision strategies—including complete gland removal—are warranted to address localized prominence, nipple protrusion, or asymmetry [17]. Therefore, accurate preoperative characterization of male breast tissue composition is instrumental not only for procedural planning and outcome prediction but also for facilitating informed discussions with patients. In our institution, this consideration underpins the routine adoption of preoperative MRI for surgical candidates with clinically significant gynecomastia, as high-resolution volumetric assessment provides objective insight into tissue composition, strengthens surgical planning, and ultimately supports more predictable outcomes. Especially within the context of modern plastic surgery’s movement toward minimally invasive and individualized interventions, such imaging-based quantification may soon become standard practice.

In designing the segmentation and volumetric analysis protocol for this study, careful consideration was given to the structural complexity, boundary ambiguity, and consistency demands inherent to male breast tissue. A high-precision modeling workflow was established, centered around the ITK-SNAP platform, which enabled the semi-automated, dual-label, and anatomically distinct segmentation of both glandular and intramammary adipose tissue. This approach represents a methodological advancement in male breast imaging: seed point initialization was coupled with active contour evolution via the Snake algorithm, allowing boundary refinement to proceed adaptively. This strategy significantly minimized operator-dependent variability while ensuring high inter-subject consistency in segmentation contours, thereby meeting the rigorous image quality demands of structural volumetric studies [18]. Real-time 3D rendering and synchronized multi-planar correction during the segmentation process further empowered operators to interactively refine boundaries in anatomically challenging regions, enhancing the accuracy and fidelity of the final models.

The successful implementation of this segmentation strategy was fundamentally enabled by the T1_FL3D_TRA_SPAIR sequence, which served as a technical backbone by providing high-resolution, high-contrast MR images. This sequence combines two advanced imaging principles: Fast Low Angle Shot 3D (FL3D), a rapid gradient echo technique, and Spectral Adiabatic Inversion Recovery (SPAIR), a fat suppression strategy optimized for spectral selectivity. FL3D employs low flip angles, short echo times, and abbreviated repetition intervals to acquire three-dimensional datasets with high in-plane resolution within clinically acceptable scan durations; in our protocol, voxels were anisotropic (0.94 × 0.94 × 5.0 mm) rather than isotropic [19]. Accordingly, high in-plane resolution—rather than isotropy—enabled reliable multiplanar review and accurate surface reconstruction, which proved critical for maintaining anatomical continuity and delineating gland–fat interfaces [20]. Moreover, FL3D is particularly advantageous in breast MRI due to its intrinsic resilience to motion artifacts caused by respiration, cardiac pulsation, or chest wall movement, thereby ensuring image stability and enhancing soft tissue clarity [21]. In this study, the use of FL3D ensured clear demarcation and homogeneous signal representation of both glandular and adipose tissues at the source image level, facilitating reliable seed placement and Snake evolution. Complementing this, SPAIR provided uniform and robust fat suppression without compromising water signal integrity [22]. Compared with conventional short inversion time recovery (STIR) or chemical shift selective methods, SPAIR offers superior tolerance to magnetic field inhomogeneities, making it particularly suited for high-susceptibility regions such as the chest and breasts [23]. Within the acquired image sets, glandular tissue—rich in water content—appeared as high signal intensity, whereas fat was effectively suppressed and rendered hypointense. This stark contrast significantly improved the label classification accuracy in ITK-SNAP and reduced the likelihood of boundary ambiguity or segmentation artifacts. The synergistic combination of FL3D and SPAIR optimized both spatial resolution and tissue contrast, enabling high-fidelity 3D reconstructions of glandular and adipose compartments. When integrated with ITK-SNAP’s semi-automated contour evolution and multi-label modeling capabilities, this protocol facilitated not only the independent identification and volumetric extraction of target structures but also their anatomically faithful visualization. As a result, the entire segmentation pipeline demonstrated excellent standardization, reproducibility, and inter-subject spatial coherence, laying a robust imaging foundation for subsequent quantitative analysis and statistical modeling.

This study provides a dual contribution: on one hand, the establishment of a reproducible MRI-based 3D segmentation and reconstruction workflow tailored for gynecomastia; on the other hand, the volumetric characterization of glandular and adipose tissues derived from this workflow. These two aspects are inseparable, as the credibility of volumetric findings relies on methodological rigor, while the volumetric distributions in turn demonstrate the workflow’s clinical applicability. To ensure robustness, consistency, and anatomical validity of the image segmentation results, this study adopted a stringent quality control framework throughout the 3D modeling process. All segmentation procedures were performed by a single, well-trained operator using a standardized workflow to minimize inter-operator variability. Key procedural elements—including seed placement, label assignment, and activation of the Snake algorithm—were executed under consistent logic. Software platform, version, and image resolution parameters were uniformly applied across all cases to mitigate systemic bias introduced by technical heterogeneity.

For seed initialization, the radius was empirically standardized between 3.0 and 6.0 voxels, a range optimized to match the in-plane spatial resolution (0.9375 mm) and slice thickness (5.0 mm) of the T1-weighted MRI images, as well as the expected anatomical dimensions of the glandular tissue. In pilot experiments, radii below 3.0 voxels frequently resulted in inadequate region growth, necessitating redundant placements, whereas values exceeding 6.0 voxels often led to overgrowth into adjacent adipose compartments. This calibrated range proved efficient and anatomically compatible during segmentation, aligning with parameter values commonly reported in 3D volumetric modeling literature [18].

To further enhance structural fidelity, each segmentation label underwent layer-by-layer inspection using ITK-SNAP’s synchronized tri-planar review function. Particular attention was paid to boundary continuity and signal transitions, especially in anatomically ambiguous zones. This post-segmentation validation ensured spatial closure and topological integrity of both glandular and adipose compartments, thereby reinforcing the accuracy of subsequent volumetric extraction and statistical measurements.

To address potential concerns regarding operator dependency and methodological reproducibility—common critiques in semi-automated segmentation workflows—the study prioritized process standardization from the outset. A detailed standard operating procedure (SOP) was developed and enforced, covering all critical steps of the segmentation pipeline. In selected cases, repeat segmentations and cross-operator validations were performed to evaluate procedural reproducibility. These methodological safeguards collectively established a traceable and replicable modeling framework that supports internal stability and external transferability.

A key methodological decision was to perform volumetry at the patient level using bilateral totals rather than per-side outputs. This choice was pre-specified to maximize measurement validity under our acquisition and segmentation conditions. First, side-specific partitioning would require an operator-defined midline in the supine orientation, where anterior chest wall flattening and a blunted intermammary sulcus provide no consistent anatomical substrate across slices; with anisotropic voxels (0.94 × 0.94 × 5.0 mm), such partitioning magnifies through-plane partial-volume and staircase artifacts. Second, our validated SOP is optimized for bilateral envelope closure with seed-based active contours and tri-planar verification; imposing post hoc left–right cuts would introduce additional manual heuristics outside the validated workflow, shifting label boundaries and degrading repeatability. Third, clinically meaningful per-side ground truth was not available: combined gland excision with liposuction does not yield side-pure, volumetrically reliable specimens (aspirate volumes are confounded by tumescent fluid and intraoperative sculpting). For these reasons, all statistical inferences were made at the patient level (bilateral totals, *N* = 52), which preserves independence, aligns with the bilateral nature of surgical planning, and—critically—avoids operator-dependent partition error that per-side outputs would introduce under the present protocol.

Despite the methodological rigor employed in segmentation and volume analysis, several intrinsic limitations and external constraints of this study warrant explicit acknowledgment. First, the MRI data were acquired using a fixed protocol on a 1.5 T SIEMENS Aera scanner, employing the default T1_FL3D_TRA_SPAIR sequence with a slice thickness of 5 mm. While this parameter setting ensures efficient scan time and spatial continuity, the relatively coarse through-plane resolution may limit the accurate delineation of small or poorly defined glandular structures. Such limitations introduce the potential for underestimation of glandular volume, particularly in cases with indistinct anatomical boundaries [24]. Nonetheless, the high in-plane resolution of 0.94 × 0.94 mm preserved boundary sharpness and enabled reliable multiplanar verification and surface reconstruction, thereby partially mitigating the volumetric distortion typically associated with anisotropic voxels. To further address this risk, the study incorporated multiple quality assurance strategies during segmentation, including synchronized multiplanar inspection, targeted seed-point refinement in critical slices, and comprehensive 3D verification of spatial closure. These measures served to minimize segmentation artifacts arising from limited z-axis resolution and to safeguard the anatomical integrity and volumetric completeness of the extracted data. Second, all imaging was performed in the supine position with an anterior body array coil, a setup intentionally adopted because it mirrors intraoperative positioning and therefore aligns closely with the aim of preoperative planning. Although this differs from the conventional prone position with a dedicated breast coil used in diagnostic breast MRI, the supine configuration was advantageous for surgical relevance and provided consistent volumetric data across patients. While direct correction factors or intraoperative validation were not applied in this study, the standardized acquisition protocol and reproducible segmentation workflow mitigate concerns about positional variability, and future investigations may further examine potential differences between supine and prone acquisitions. Third, the study population consisted exclusively of bilateral gynecomastia (GM) patients scheduled for surgical intervention, a cohort characterized by prominent clinical symptoms, substantial structural hypertrophy, and documented preoperative imaging. As such, the derived tissue composition patterns and statistical associations are most applicable to diagnostic imaging and preoperative planning scenarios in symptomatic surgical candidates. Extrapolation of these findings to broader GM populations—including pubertal self-resolving cases, asymptomatic individuals, or patients with unilateral involvement—should be approached with caution, as structural profiles may differ substantially and limit statistical comparability. In addition, intraoperative excised tissue weights or pathology-based volumetric validation were not available in this cohort, since glandular excision was frequently combined with liposuction, and aspirated fat volumes are confounded by irrigation fluid and sculpting. As a result, reliable side-specific or specimen-based volumetric correspondence could not be established. This limitation does not undermine the objectives of the present work, which were confined to preoperative structural modeling and descriptive quantification. Nonetheless, future studies incorporating dedicated validation subsets—such as cases with isolated glandular excision or prospectively recorded specimen weights—will be essential to further strengthen the translational accuracy of MRI-based volumetry.

From a technical standpoint, the segmentation pipeline developed here features well-defined parameters, scalable outputs, and a modular architecture that supports cross-institutional replication and integration into machine learning workflows. These attributes position the current framework as a foundational tool for establishing a standardized imaging database of male breast morphology. Coupled with artificial intelligence, the model holds potential for training automated classifiers capable of preoperative tissue categorization, ultimately enabling more accurate and individualized clinical decision-making. Future iterations may also integrate imaging data with endocrine profiles, genotypic information, and metabolic parameters to build multidisciplinary predictive models for gynecomastia risk and progression.

## 5. Conclusions

This study establishes a reproducible workflow for preoperative MRI-based three-dimensional segmentation and reconstruction of glandular and adipose tissues in male gynecomastia. By integrating fat-suppressed T1-weighted imaging with active contour modeling, the method achieves precise tissue delineation, ensures anatomically continuous reconstruction and yields consistent volumetric measurements. The resulting models offer high-resolution visualization of tissue architecture, facilitating objective structural assessment to support individualized surgical planning.

## Data Availability

The data that support the findings of this study are not publicly available due to privacy restrictions. De-identified data can be made available upon reasonable request to the corresponding author and subject to approval by the institutional ethics committee.
